# Erythropoietin Regulates Transcription and YY1 Dynamics in a Pre-established Chromatin Architecture

**DOI:** 10.1016/j.isci.2020.101583

**Published:** 2020-09-20

**Authors:** Andrea A. Perreault, Jonathan D. Brown, Bryan J. Venters

**Affiliations:** 1 Chemical and Physical Biology Program, Vanderbilt University, Nashville, TN 37232, USA; 2 Division of Cardiovascular Medicine, Department of Medicine, Vanderbilt University Medical Center, Nashville, TN 37232, USA; 3 Molecular Physiology and Biophysics, Vanderbilt University, Nashville, TN 37232, USA

**Keywords:** Molecular Biology, Developmental Biology, Genomics

## Abstract

The three-dimensional architecture of the genome plays an essential role in establishing and maintaining cell identity. However, the magnitude and temporal kinetics of changes in chromatin structure that arise during cell differentiation remain poorly understood. Here, we leverage a murine model of erythropoiesis to study the relationship between chromatin conformation, the epigenome, and transcription in erythroid cells. We discover that acute transcriptional responses induced by erythropoietin (EPO), the hormone necessary for erythroid differentiation, occur within an invariant chromatin topology. Within this pre-established landscape, Yin Yang 1 (YY1) occupancy dynamically redistributes to sites in proximity of EPO-regulated genes. Using HiChIP, we identify chromatin contacts mediated by H3K27ac and YY1 that are enriched for enhancer-promoter interactions of EPO-responsive genes. Taken together, these data are consistent with an emerging model that rapid, signal-dependent transcription occurs in the context of a pre-established chromatin architecture.

## Highlights

• EPO induces rapid RNA Pol II response at a key subset of genes
• YY1 is redistributed in the genome following 1 h EPO stimulation
• CTCF and YY1 bind different locations pre and post 1 h EPO stimulation
• E-P loops mediated by H3K27ac are largely invariant in response to EPO

## Graphical Abstract

**Figure:**
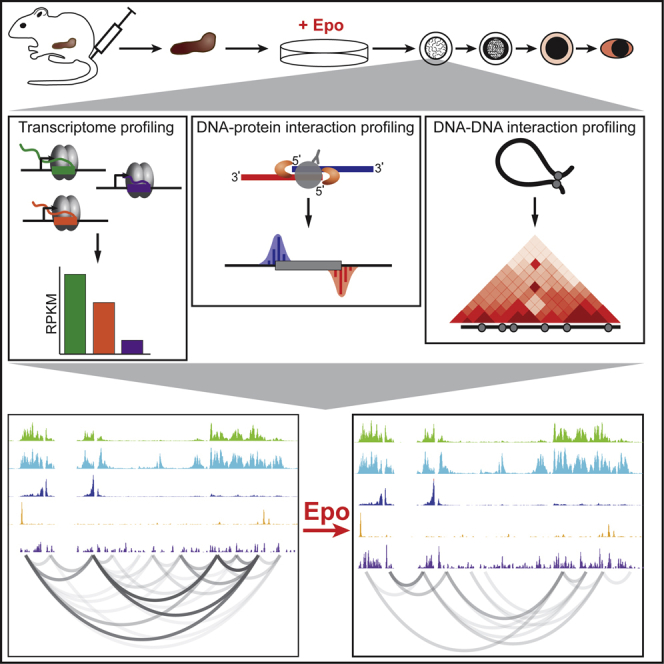

## Introduction

Transcription control is a primary mechanism for regulating gene expression in eukaryotes. Three major steps exist in the transcription cycle: (1) preinitiation complex (PIC) formation, (2) pause release of RNA polymerase II (Pol II) to productive elongation, and (3) transcription termination (Liu et al., 2015). Multiple mechanisms exist to regulate each step, thereby providing precise control over the magnitude and kinetics of transcription and global gene expression. Promoter proximal pausing is one such mechanism and is recognized as a general feature of transcription at many eukaryotic genes. Specifically, there is a prominence of paused Pol II at signal-responsive genes, which serves to prime these genes for rapid transcription in response to environmental stimuli (Adelman et al., 2009; Gaertner et al., 2012; Danko et al., 2013). Transcription factor (TF)-bound enhancers activate Pol II, acting as an additional mechanism in regulating transcription (Heintzman et al., 2009) and defining cell identity (Ernst et al., 2011; Zhu et al., 2013). Athough chromatin state maps are useful to assign enhancers to target genes based on distance from promoters, proximity analysis is overly simplistic with respect to the true gene regulatory environment (Mora et al., 2016; Yao et al., 2015; Rao et al., 2014).

More recently, high-resolution maps of the three-dimensional (3D) genome have revealed that enhancers exhibit long-range control of transcription. Structural proteins, such as CCCTC-binding factor (CTCF) and Yin Yang 1 (YY1), tether distal TF-bound enhancers to their target gene promoters. CTCF is an evolutionarily conserved zinc finger that co-localizes with cohesin (Phillips and Corces, 2009). Together, these two factors establish and maintain chromatin loops (Sanyal et al., 2012; Rao et al., 2017; Ren et al., 2017). Assays that can map chromatin contacts, such as HiC, have revealed that the genome is organized into topologically associated domains (TADs), which are demarcated by CTCF (Lieberman-Aiden et al., 2009; Dixon et al., 2012) and are largely consistent between cell types (Phillips and Corces, 2009; Ong and Corces, 2014; Arzate-Mejia et al., 2018). These large domains can be further separated into subTADs that contain higher contact frequencies between regions of the genome, many of which are not limited to one-to-one interactions (Li et al., 2012; Sanyal et al., 2012; Fullwood et al., 2009; Dowen et al., 2014; Ji et al., 2016). Together, these findings demonstrate CTCF's function as structural foci for chromatin organization, whereby Pol II can selectively target cell-type-specific genes for transcription through interactions with looping factors and enhancers.

Other TFs, such as YY1, are specifically enriched at chromatin loops that connect enhancers to promoters of actively transcribed genes (Weintraub et al., 2017). YY1 is a ubiquitously expressed zinc-finger TF that plays an important role in cellular differentiation (Kleiman et al., 2016; Beagan et al., 2017). Deletion of YY1-binding motifs at gene promoters in mouse embryonic stem cells (ESCs) reduced contact frequency between individual promoters and enhancers, and variably reduced mRNA levels (Weintraub et al., 2017). These data provide evidence for an essential role of YY1 in controlling gene expression by facilitating enhancer-promoter (E-P) interactions.

Erythropoiesis has been a useful model system for understanding the interplay between Pol II dynamics (Johnson et al., 2002; Sawado et al., 2003), enhancer activity (Reik et al., 1998), and 3D genome structure (Tolhuis et al., 2002; Chien et al., 2011; Deng et al., 2012; Bartman et al., 2016) during cellular differentiation. Indeed, we have previously characterized the genome-wide enhancer landscape in proerythroblasts (ProEBs) in response to erythropoietin (EPO) (Perreault et al., 2017), the hormone that is required for terminal erythroid differentiation (Koury and Bondurant, 1988, 1990). However, the manner by which EPO signaling shapes the 3D genome and specific chromatin interactions remains poorly understood. In addition, although CTCF occupancy and function has been assessed in erythroid cells (Hanssen et al., 2017; Hsu et al., 2017; Lee et al., 2017), the YY1-binding locations in erythroid cells are not known, resulting in a knowledge gap in uncovering the role of important TFs controlling E-P interactions and overall chromatin architecture during erythropoiesis.

**Figure 1:**
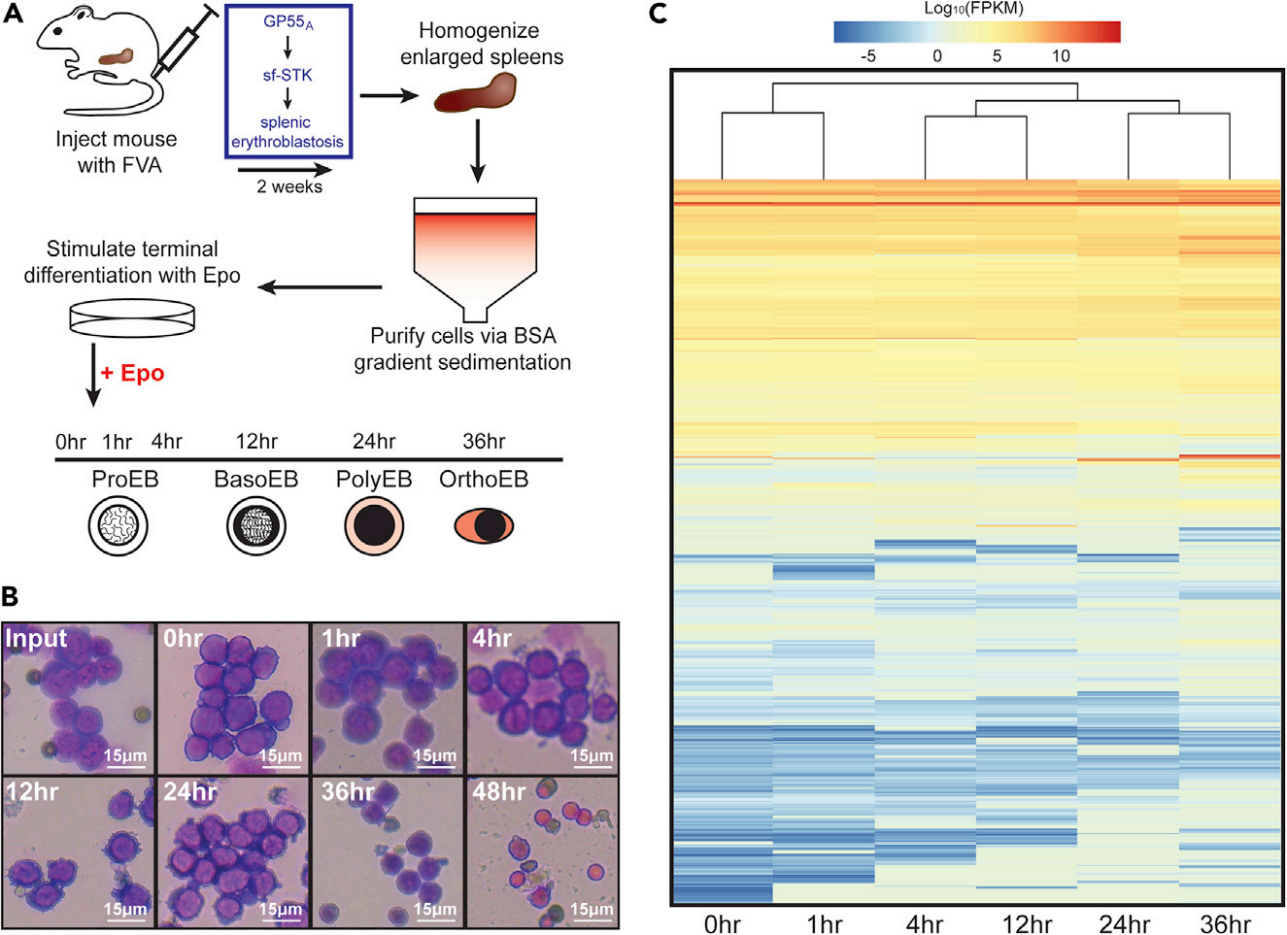
The FVA Murine System Faithfully Recapitulates Erythroid Differentiation during Erythropoiesis (A) The workflow for generating and isolating highly purified EPO-responsive ProEBs from a mouse injected with the Friend virus that induces anemia (FVA). (B) Microscopy images highlighting morphological changes of ProEBs isolated using the FVA system during differentiation. (C) Heatmap of RNA-seq gene expression through erythroid differentiation.

**Figure 2:**
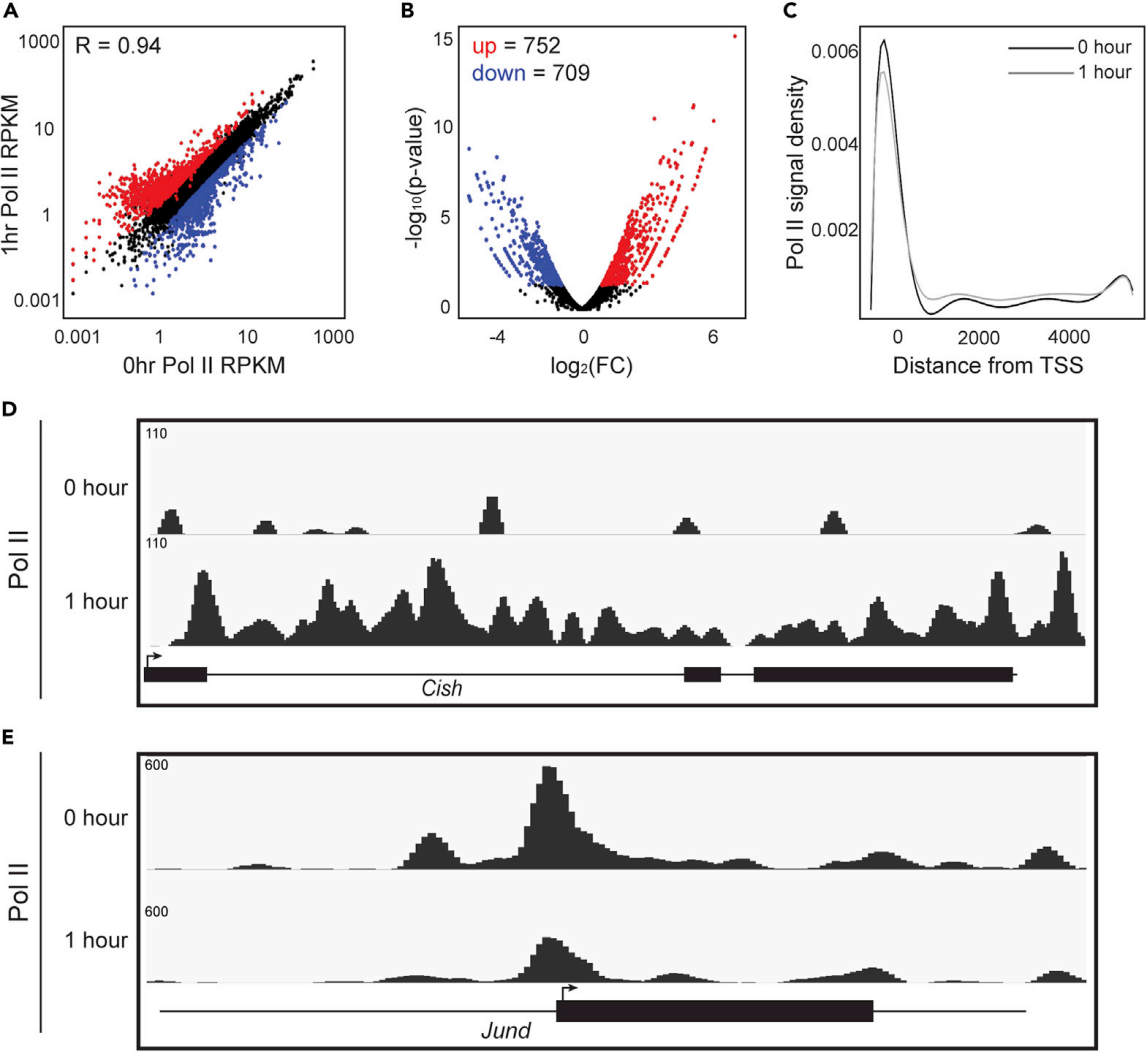
EPO Stimulation Results in Acute Transcriptional Changes in Proerythroblasts (A) Scatterplot comparing Pol II RPKM before and after 1 h EPO stimulation. Pearson's correlation value R = 0.94. (B) Volcano plot showing significant (p value < 0.05) differential occupancy of increased (red) and decreased (blue) Pol II after 1-h EPO stimulation. (C–E) (C) Metagene plot comparing the position of Pol II peaks relative to transcription start site (TSS) (paired Wilcoxon ranked-sign test, p = 4.882 × 10^−11^). Genome browser view of ChIP-exo signal for Pol II at the up-regulated *Cish* locus (D) and down-regulated *Jund* locus (E).

**Figure 3:**
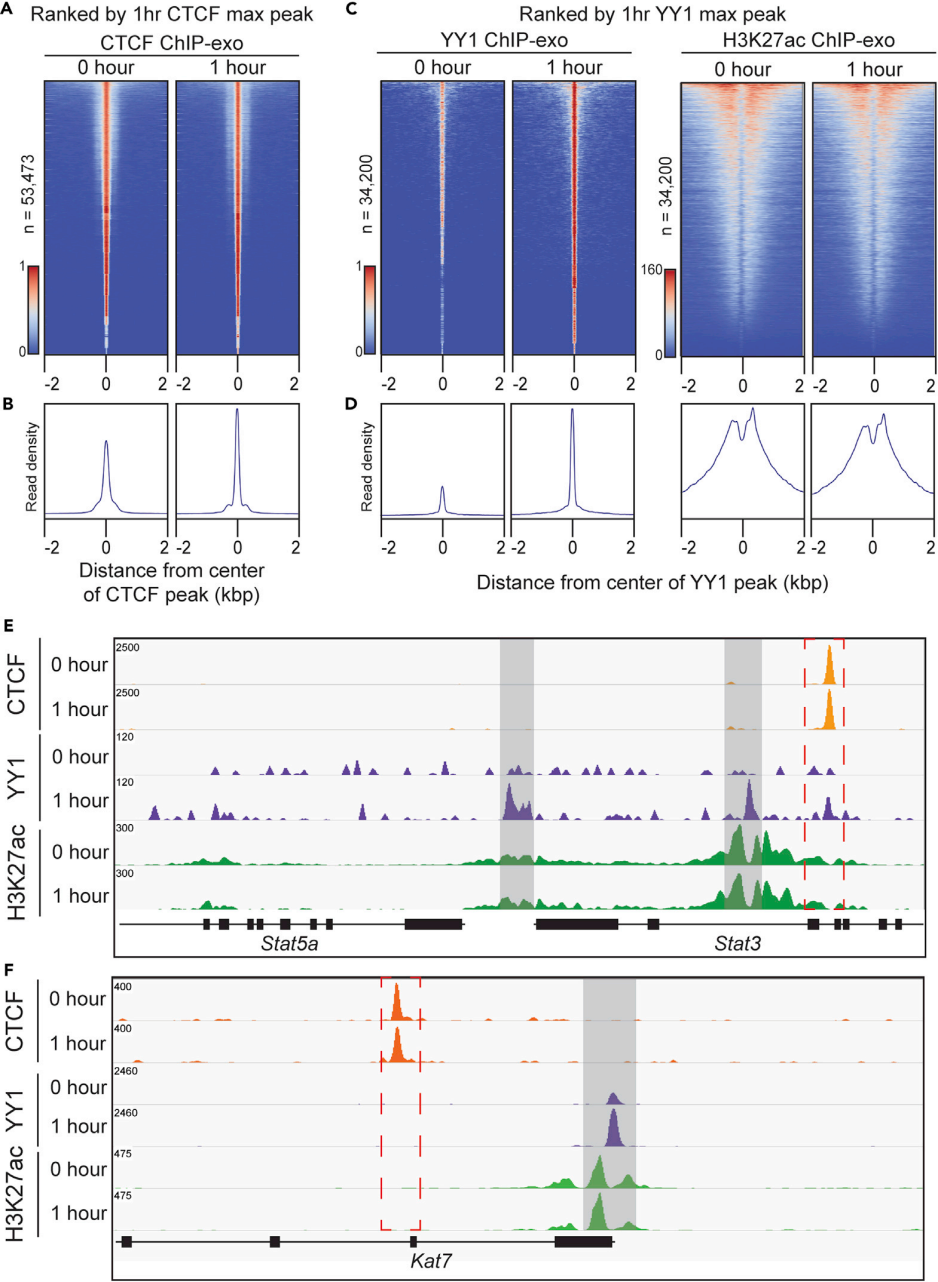
EPO Dynamically Regulates YY1 Occupancy Genome-wide (A) Heatmap of CTCF peaks pre- and post-EPO stimulation, ranked by 1 h CTCF max peak. (C) Heatmap of YY1 and H3K27ac peaks pre- and post-EPO stimulation, ranked by 1 h YY1 max peak. (B and D) Composite plots below each heatmap quantifying the normalized tag density. (E and F) Representative genome browser view of CTCF, YY1, and H3K27ac occupancy in response to EPO stimulation, highlighted in light gray bars and red dashed box.

**Figure 4:**
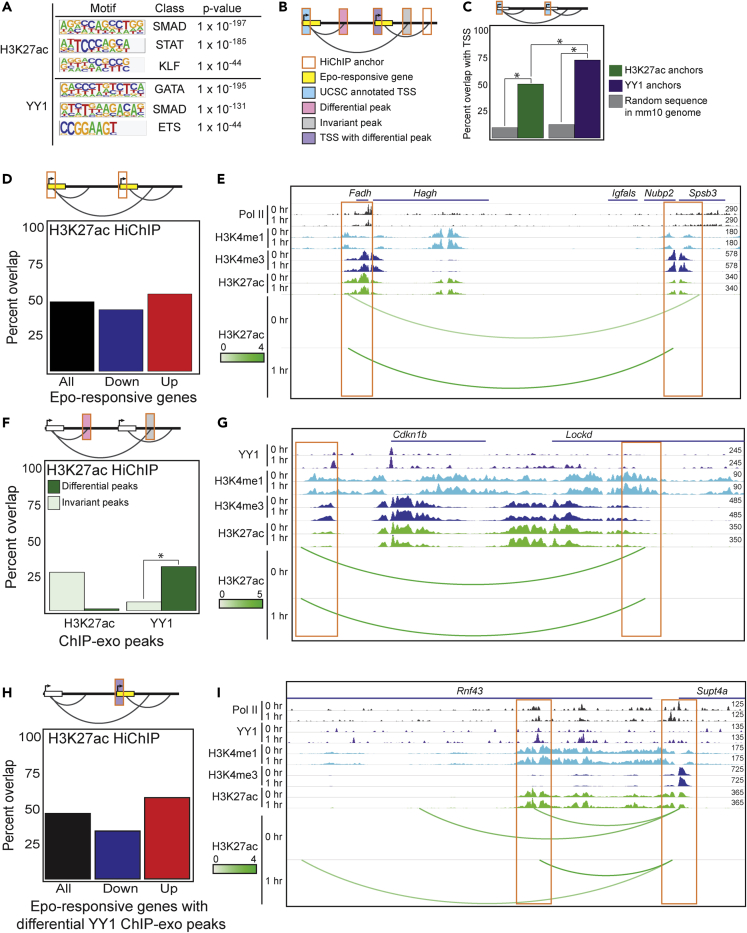
EPO Regulates Transcription in a Pre-established Chromatin Conformation (A) TF-binding motifs overrepresented in HiChIP loop anchors. (B) A schematic of chromatin features. (C) Proportion of HiChIP interactions with UCSC-annotated TSS within anchor regions compared with random sequences in mm10 genome (gray bars) (∗p < 0.0001). The hypergeometric test was applied to compare HiChIP anchors found in annotated TSSs to expected ratios. The chi-squared test was applied to compare TSS occupancy between H3K27ac and YY1 anchors, as well as comparing HiChIP anchors to randomly generated sequences in the mouse genome. (D) Proportion of HiChIP interactions with promoters of EPO-responsive genes within H3K27ac HiChIP anchor regions. (E) Representative genome browser view of overlap described in (D) with anchor regions highlighted in orange boxes. (F) Proportion of HiChIP interactions with differential H3K27ac or YY1 ChIP-exo peaks within H3K27ac HiChIP anchor regions. Dark green bars represent differential peaks, and light green bars represent invariant peaks (∗p < 0.0001). The chi-squared test was applied to compare YY1 differential and invariant peaks in H3K27ac anchors. (G) Representative genome browser view of overlap described in (F) with anchor regions highlighted in orange boxes. (H) Proportion of HiChIP interactions with differential YY1 peaks at promoters of EPO-responsive genes within H3K27ac HiChIP anchor regions. (I) Representative genome browser view of overlap described in (H) with anchor regions highlighted in orange boxes.

To address this critical gap in understanding, we leveraged a murine model system to study synchronous erythroid maturation *ex vivo* in response to EPO stimulation (Figure 1) (Bondurant et al., 1985; Koury et al., 1984; Sawyer et al., 1987). Here, we demonstrate that EPO stimulates rapid transcriptional changes in ProEBs after 1 h (Figure 2). During this time, YY1 occupancy is dynamically redistributed, as opposed to CTCF, which remains unchanged (Figure 3). Moreover, there is little overlap in the regions bound by these structural TFs. Using HiChIP, we determined the chromatin contacts mediated by H3K27ac and YY1 genome-wide. We discover that a subset of these chromatin interactions remains invariant during EPO signaling, facilitating unique E-P interactions during EPO-mediated transcriptional regulation (Figure 4).

## Results

### EPO Induces Terminal Erythrocyte Differentiation through Changes in Gene Expression

The anemia-inducing strain of the Friend virus (FVA) system enables us to investigate the temporal dynamics of gene regulation and genome architecture in response to hormone stimulation. In this model, systemic treatment of mice with FVA induces ProEB proliferation in the spleen. After 14 days, large quantities of lineage-committed ProEBs can be isolated and purified. Stimulation of ProEBs with the hormone EPO in an *ex vivo* culture system induces synchronous terminal differentiation into mature erythrocytes over a 48 h period (Figure 1A) (Sawyer et al., 1987).

Using this model system, we observed a predictable shift in size and shape of maturing erythroid precursors during erythropoiesis, as visualized by light microscopy of H&E-stained cells. Before purification, the cells appear heterogeneous (Figure 1B, Input). After purification, a uniform population of ProEBs is obtained, evident as large, round cells. This morphological stage persists until approximately 12 h after the start of EPO stimulation (Figure 1B). After 24 h of EPO, cells form polychromatic erythroblasts (PolyEBs), characterized by the accumulation of hemoglobin, which coincides with the continued increase in globin gene expression. Finally, after 48 h of *ex vivo* culture in EPO, the cells have terminally differentiated into reticulocytes, marked by high hemoglobin production and nucleus extrusion (Figure 1B, 48hr).

Having established the kinetics of this model system, we next examined how differentiation impacts the erythroid gene expression program over time. RNA sequencing (RNA-seq) at six time points demonstrated both up- and down-regulation of genes when compared with EPO-naive cells (Figure 1C, Table S1). Overall, approximately 12,000 genes had differential expression during the entire 48 h time course of erythropoiesis, with 8,105 of those genes significantly differentially expressed (adjusted q-value < 0.05). More specifically, there were 685 genes that significantly changed expression after only 1 h of EPO (adjusted q-value < 0.05). This transcriptomic dataset highlights the large changes in gene expression that accompany the morphological shifts occurring during erythropoiesis.

### EPO Activates Rapid Transcriptional Changes in ProEBs

The progressive changes in gene expression observed by RNA-seq reflect global transcriptional responses. We set out to investigate the immediate transcriptional response to EPO in purified ProEBs. To assess the acute effect of hormone stimulation on transcription, we performed chromatin immunoprecipitation (ChIP)-exo for RNA polymerase II (Pol II) before and after 1 h of EPO stimulation. Overall, Pol II occupancy is highly correlated when comparing ChIP-exo signal pre- and post-EPO stimulation in this short time frame (Figure 2A, Table S2). This result indicates that global Pol II occupancy does not change at the majority of transcribed genes after 1 h. However, analysis of fold change of Pol II signal in these conditions did identify significant differential occupancy of Pol II at a smaller subset of genes (p < 0.05) (Figure 2B). We detected both significantly increased (n = 752, red) and decreased (n = 709, blue) Pol II signal at these EPO-responsive loci, indicating a set of genes that are regulated at the transcriptional level by EPO after only 1 h.

Overall, gene expression as measured by RNA-seq and transcription as measured by Pol II ChIP-exo are not in concordance with one another, specifically when investigating after 1 h EPO stimulation. There are 450 unique differentially expressed genes with consistent Pol II occupancy at promoters. Gene ontology (GO) analysis of these genes reveals an enrichment for genes involved in regulation of erythrocyte development, tyrosine phosphorylation of STAT protein, and response to hormonal stimulus, among others. This supports the role of the identified differentially expressed genes in response to EPO during erythroid maturation. However, when investigating stably expressed genes that have differential Pol II occupancy at promoters, there is no significant enrichment for specific biological processes. The explanation of the discordance of these features represents an area of gene regulation that requires further investigation.

Rapid transcriptional induction may reflect PIC assembly, pause release of Pol II, or a combination of these steps. To investigate the mechanism of EPO-regulated transcription, we next mapped Pol II signal at transcriptional start sites (TSS) or gene bodies of all induced genes. This approach identified that Pol II is more abundant at the TSS of genes before EPO (Figure 2C). After 1 h of EPO stimulation, Pol II transitions beyond the TSS into the gene body, indicative of pause release of Pol II at induced genes.

The dynamics of increased Pol II occupancy can be visualized at an exemplary locus of cytokine-inducible SH2-containing protein (*Cish*) (Figure 2D). *Cish* is a known target of the JAK-STAT signaling pathway, which is directly activated by EPO (Matsumoto et al., 1997; Rascle and Lees, 2003). We also observed down-regulation of transcription, including the *Jund* gene (Figure 2E). *Jund* is a component of the AP1 complex, which regulates response to cytokines, growth factors, stress, and infections in a variety of cellular contexts (Karin et al., 1997; Hernandez et al., 2008). *Jund*, along with other members of the Jun family, has been found to prevent differentiation in murine erythroleukemic cells (Prochownik et al., 1990), highlighting the critical need to down-regulate this gene during erythropoiesis. The importance of *Cish* and *Jund* in differentiation provides specific examples of the biological significance of the early EPO-mediated transcriptional responses described here.

### EPO Dynamically Regulates YY1 Occupancy Genome-wide

Signal-dependent activation of Pol II is accompanied by alterations in chromatin organization. However, the impact of EPO on chromatin structure and function during erythropoiesis is not well understood. To begin addressing this question, we first examined the genome-wide occupancy patterns of CTCF and YY1, two TFs known to play key roles in genome organization and gene regulation. CTCF occupancy did not change between pre and post 1 h EPO treatment, as demonstrated by the comparison of global enrichment analysis at each time point (Figures 3A and 3B, Tables S2 and S3). The stable CTCF-binding reflects the fact that the cells in each treatment group are lineage-committed ProEBs (Figures 1A and 1B). Thus, the invariant occupancy of CTCF observed is consistent with recent studies demonstrating that CTCF decreases variability in gene expression and thereby functions to maintain an established cell state (Ren et al., 2017).

In contrast to CTCF, YY1 is rapidly redistributed in the genome following 1 h EPO stimulation (Figures 3C and 3D, Tables S2 and S3). Of the 103,705 YY1 peaks found pre-EPO stimulation, only 4,843 peaks were found at the same regions after EPO stimulation. In EPO-naive ProEBs, the majority of YY1 localized to intergenic regions (blue region, 48%, Figure S1A). However, this localization shifted to intronic sites after EPO (gray region, 42%, Figure S1A). Notably, YY1 ChIP-exo signal at TSSs significantly increased from 5% to 17% following EPO (Figure S1A, hypergeometric test: p value <0.0001). These results specifically elaborate on the shifting genomic regions that YY1 binds pre and post 1 h of EPO stimulation. Comparison of CTCF and YY1 also revealed minimal overlap in localization of these two factors pre- and post-EPO stimulation, 7% and 5%, respectively (Figure S1B, hypergeometric test: p value < 0.0001). These results suggest that the chromatin domains established by CTCF and YY1 are distinct and these structural proteins have unique functions during EPO-dependent gene regulation in ProEBs.

In addition, ranking of H3K27ac signal by YY1 enrichment demonstrated that H3K27ac signal did not change in an appreciable manner compared with YY1 after EPO. This suggests that a subset of YY1 sites were more dynamic than H3K27ac, which is commonly used to identify active enhancers in the genome (Figures 3C and 3D).

At the *Stat3* gene, an exemplary locus of EPO-mediated transcription, a strong CTCF peak is evident pre-EPO and does not change after EPO (Figure 3E). At the same *Stat3* locus, YY1 occupancy increases at multiple *de novo* binding sites, as well as one site that overlaps with CTCF. Similar changes in YY1 can be visualized at another EPO-responsive gene, *Kat7* (Figure 3F). These specific loci illustrate how EPO induces a dynamic change in YY1 occupancy at a subset of genes relevant to signal transduction and chromatin modification during erythropoiesis.

### EPO Regulates Transcription in a Pre-established Chromatin Conformation

Our discovery that YY1 rapidly redistributes in the ProEB genome following EPO stimulation prompted us to explore the role of YY1 and H3K27ac in chromatin organization. To accomplish this goal, we identified chromatin interactions using HiChIP (Table S4). HiChIP is a chromosome conformation capture assay that maps chromatin interactions between specific factors genome-wide (Mumbach et al., 2016). Chromatin interactions and high likelihood chromatin loops were identified using hichipper program (Tables S5 and S6) (for details, see Transparent Methods) (Lareau and Aryee, 2018b). We examined the global chromatin interactions mediated by YY1 or H3K27ac in EPO-stimulated ProEBs using Juicer (Durand et al., 2016) (Figures S2A–S2L). H3K27ac and YY1 anchors were approximately 4 kilobases (kb) on average both before and after EPO (Figure S3A). In addition, the average interaction lengths between either H3K27ac or YY1 anchors were approximately 317 kb, with no evident change in loop length in response to EPO treatment (Figure S3B).

To investigate the biological significance of these interactions in ProEBs, we first conducted unbiased, *de novo* motif discovery analysis using DNA sequences from all HiChIP anchor regions. Strikingly, we identified an enrichment of consensus motifs of multiple TF families known to regulate specification of the erythroid lineage, including STAT (Kisseleva et al., 2002; Watowich, 2011), KLF (Miller and Bieker, 1993; Cantor and Orkin, 2002; Kang et al., 2015), and GATA (Weiss and Orkin, 1995; Cantor and Orkin, 2002; Lentjes et al., 2016) (Figure 4A). In addition, auxiliary factors, such as SMAD and ETS, aid in the maintenance of gene expression and lineage commitment, respectively (Schmerer and Evans, 2003; Pimkin et al., 2014; Schuetze et al., 1993). The enrichment of these consensus motifs within YY1 and H3K27ac HiChIP anchor regions suggests functional coupling between erythroid TFs and chromatin conformation during erythropoiesis.

Overall, we identified 151,468 H3K27ac- and 138,583 YY1-mediated chromatin contacts in ProEBs using diffloop (for details, see Transparent Methods) (Lareau and Aryee, 2018a). The majority of these loops had a score less than 5 (Figures S3C and S3D), indicating that weak interactions predominate pre- and post-EPO stimulation. Using fold change of loop scores as a metric for altered chromatin organization, we classified interactions as variant or invariant. We identified 109,390 invariant H3K27ac-mediated and 5,414 invariant YY1-mediated contacts. Interestingly, 99% of H3K27ac and 97% of YY1 strong contacts were invariant.

As strong contacts are likely to be more robust than other contacts, we therefore focused further investigation on invariant loops to gain insight into the relationship between constant chromatin organization and transcriptional response, as has been conducted in recent literature (Jin et al., 2013; D'Ippolito et al., 2018; Ray et al., 2019). We reasoned that a better understanding of the interactions between enhancers and promoters might provide new insights into how EPO regulates transcription. We classified 23,423 H3K27ac and 21,777 YY1 E-P loops using diffloop (Lareau and Aryee, 2018a), which were further delineated into 16,698 and 1,444 invariant E-P loops for H3K27ac and YY1, respectively. The majority of E-P loops are found in intronic regions and did not shift location in the genome based on variance or factor (Figure S3E).

To gain insights into how E-P loops are involved in transcriptional regulation, we aimed to investigate the interplay of chromatin interactions, gene expression, and TF binding. The schematic in Figure 4B depicts these features.

We first quantified the proportion of HiChIP anchors (H3K27ac or YY1) of invariant E-P contacts that map to UCSC-annotated TSSs in the mm10 reference genome (Figure 4C). We would expect 50% of the anchors to be found in TSS regions if each promoter was connected to one enhancer. Indeed, 50% of the H3K27ac anchors were observed in annotated TSSs (Figure 4C, hypergeometric test: p < 0.0001). In contrast to H3K27ac, 73% of YY1 anchors overlapped with annotated TSSs, indicating that more YY1 anchors are at promoters compared with enhancer regions (Figure 4C, hypergeometric test: p < 0.0001). These results suggest that a single enhancer could regulate the transcription of multiple target genes in chromatin interactions mediated by YY1. The difference in TSS occupancy between H3K27ac and YY1 anchors is significant (chi-squared: p < 0.0001), indicating that H3K27ac and YY1 are differentially mediating E-P interactions and their connectivity. As an additional validation, we randomly generated sequences of the mouse genome and overlapped these with anchor regions (gray bars, Figure 4C, chi-squared: p < 0.00001), which supports the conclusion that annotated TSSs are more likely to be found at loop anchors than expected due to chance.

Given that E-P interactions overlap with annotated promoter regions, we next examined the relationship between these contacts and the associated changes in transcriptional response to EPO, as described in Figure 2. Focusing only on EPO-responsive genes (n = 1,462), we identified a higher proportion of overlap for H3K27ac anchors at promoters of EPO-responsive genes when compared with YY1 anchors, 50% (Figure 4D) and 6% (Figure S4A), respectively. We also examined this relationship as a function of genes up- (n = 752, red) or down-regulated (n = 709, blue) by EPO. As expected, fewer promoters in EPO down-regulated genes overlapped with H3K27ac anchors. The persistence of H3K27ac in down-regulated promoters is consistent with prior work demonstrating that loss of H3K27ac signal at enhancers and promoters can lag behind a decrease in transcription (Brown et al., 2014). The majority (75%) of strong H3K27ac loops, however, were found at genes that were not responsive to EPO, suggesting that strong, invariant interactions sustain transcription during response to external stimulation. Figure 4E shows a representative example of this overlap at the *Fadh1* gene, which is intricately involved in mitochondrial activity and metabolism (Weiss et al., 2018). Mitochondrial biogenesis is activated by EPO and is therefore highly regulated during erythropoiesis (Carraway et al., 2010; Liu et al., 2017). These data reveal that H3K27ac-mediated loops that are weak or moderate in strength are connected to EPO-induced transcriptional response.

We were surprised that H3K27ac anchors overlapped at promoters of EPO-responsive genes more than YY1 anchors, given that YY1 genome occupancy was more dynamic (Figure 3). To resolve this apparent paradox, we first examined the relationship between differential H3K27ac or YY1 occupancy and invariant chromatin contacts. With this approach, we detected significant enrichment for differential YY1 ChIP-exo peaks at invariant H3K27ac anchors (Figure 4F, chi-squared: p < 0.0001). In contrast to this result, invariant H3K27ac loops were associated with invariant H3K27ac ChIP-exo peaks (Figure 4F). An example of this relationship can be observed at the *Cdkn1b and Lockd* loci, two genes that regulate exit of erythroid precursors from the cell cycle, a required step in differentiation (Paralkar et al., 2016) (Figure 4G). Similarly, anchors of invariant YY1 chromatin interactions were enriched at loci with differential YY1 and invariant H3K27ac ChIP-exo peaks (Figure S4B). This suggests that although certain factors, like YY1, are more dynamic than others, like H3K27ac, these features do not necessarily indicate the variance of the loops they mediate.

We then wanted to test if YY1 differential occupancy at promoters was related to transcriptional response to EPO. Indeed, this analysis revealed that H3K27ac anchors were found at promoters of EPO-responsive genes with differential YY1 ChIP-exo peaks (Figure 4H). This supports the idea that invariant chromatin interactions are facilitative environments for transcriptional and epigenetic response to hormone stimulation. A representative example of this overlap is shown at the *Supt4a* gene, which encodes the SPT4 protein, a component of the DSIF elongation complex (Schneider et al., 2006; Crickard et al., 2017), implicating this locus in transcriptional regulation. By contrast, YY1 anchors were not enriched for differential YY1 peaks or EPO-responsive genes to the same degree as H3K27ac anchors (Figure S4C). This suggests that H3K27ac and YY1 regulate chromatin architecture and therefore gene regulation through different mechanisms. Together, these results support a model whereby EPO induces dynamic transcription and TF binding within a pre-established chromatin context.

## Discussion

The findings presented here examining erythroid differentiation in response to EPO are consistent with an emerging paradigm that signal-dependent transcriptional responses occur within a pre-established chromatin landscape identified using HiC methodologies. For example, TNFalpha-responsive enhancers in human fibroblasts were already in contact with their target promoters before signaling. These results suggest a model in which signal-responsive TFs bind to enhancers to function within a pre-established chromatin architecture (Jin et al., 2013). Glucocorticoid treatment in human A549 cells revealed that glucocorticoid receptor binding to the genome did not promote new chromatin contacts, but instead induced changes in existing interactions to regulate transcription (D'Ippolito et al., 2018). HiC analysis in *Drosophila* S2 and human K562 cells identified that no global changes in TADs emerged after heat shock treatment, despite changes in TF binding and induction of heat shock response genes (Ray et al., 2019). Finally, capture HiC and 4C experiments in ESCs have provided evidence that hardwired chromatin interactions provide an environment for TF binding and enhancer activation that facilitates a rapid transcriptional response to signaling in neuronal development (Atlasi et al., 2019). Unlike these studies, our study employed the HiChIP assay to define the genome-wide contacts mediated by specific factors, namely, H3K27ac and YY1. These H3K27ac and YY1 HiChIP contacts revealed a subset of invariant chromatin loops that connect enhancers and EPO-regulated genes, thereby refining the E-P connectome in erythroid cells. These chromatin interactions provide important insights to conformational features, such as enhancer skipping and promoter-promoter interactions, which cannot be determined using 1D chromatin features (Mumbach et al., 2017). Future work will investigate these conformation features to evaluate previously identified E-P interactions in ProEBs (Perreault et al., 2017).

Given that CTCF domains shift during development (Nora et al., 2017), we originally hypothesized that EPO would induce changes to CTCF occupancy and subTAD organization. However, CTCF occupancy did not change but instead decreased after EPO, supporting the idea of selective pruning of CTCF-binding sites during differentiation (Beagan et al., 2017). In contrast, YY1 did redistribute dynamically in the genome within 1 h of EPO stimulation, suggesting a more critical role for YY1 in chromatin organization in early erythroid maturation. These data are consistent with studies identifying YY1's role in E-P loops and transcriptional activation (Weintraub et al., 2017). Given that YY1 was more dynamic than H3K27ac occupancy, we speculate that combining H3K27ac and YY1 occupancy data may assign enhancers to target genes with more accuracy than H3K27ac alone. This concept will require additional studies.

Not surprisingly, we did observe dynamic changes in Pol II occupancy in response to EPO at a subset of genes both significantly up- and down-regulated. These results are consistent with a growing body of work identifying paused Pol II at signal-responsive genes. It has been proposed that this state of Pol II enables rapid transcriptional response to environmental stimuli. For example, in *Drosophila* S2 cells stalled Pol II was strongly enriched at genes that are induced by multiple signaling pathways involved in regulating development, cell differentiation, and cell communication (Muse et al., 2007). In addition, study of murine macrophage cell lines identified an accumulation of paused Pol II at the *TNFalpha* gene in quiescent cells before induction of the gene by inflammatory cytokines (Adelman et al., 2009).

There are still several aspects of EPO's impact on transcription and chromatin structure that remain unanswered. We identified discordance between dynamic YY1 binding measured by ChIP-exo and invariant YY1-mediated interactions determined by HiChIP. The majority of YY1 HiChIP interactions had weak scores (scores <5, Figure S3D), despite abundant YY1 binding in the genome. This suggests that the overall abundance of YY1 does not necessarily indicate the strength of the loop it mediates. It is possible that YY1-binding locations are establishing chromatin contacts that will gain strength over time, and therefore delineate cell-type-specific interactions more decisively as maturation continues. In addition, we expected Pol II ChIP-exo differential peaks to be found at gene promoters that exhibited differential expression as measured by RNA-seq. However, we only detected a small overlap in the gene promoters where this was the case. It is likely that steady-state gene expression measured by RNA-seq lags behind rapid transcriptional responses assessed by Pol II ChIP-exo. Future studies will investigate this relationship between Pol II occupancy and gene expression across the entire period of erythroid maturation in the FVA model.

Taken together, the results presented here integrate epigenetic and transcriptional profiles with genome-wide HiChIP datasets to describe how hormone stimulation regulates erythroid differentiation. We demonstrate that dynamic features occur within static chromatin interactions. Future work will focus on integrating changes in Pol II, CTCF, H3K27ac, and YY1, as well as the chromatin contacts they mediate, during erythropoiesis with the goal of understanding how the 3D genome influences transcription and dynamic gene regulatory programs during erythroid maturation. This knowledge will have a significant impact on our understanding of the interplay between signal-dependent transcription and chromatin architecture.

### Limitations of the Study

Although the FVA system provides the ideal model system to study isolated, pure populations of cells during erythroid differentiation, the presented study investigates the first hour of erythropoiesis. There are transcriptional and epigenetic dynamics during this narrow time frame, but the large-scale changes that occur during erythroid maturation remain to be investigated. Mainly, the invariant chromatin structure described here may be a unique feature of the ProEBs that have been stimulated with EPO for 1 h. In addition, the discordance of gene expression as measured by RNA-seq and the transcriptional responses assessed by Pol II ChIP-exo may be a result of the short stimulation time studied here.

### Resource Availability

#### Lead Contact

Further information and requests for resources and reagents should be directed to and will be fulfilled by the Lead Contact, Andrea Perreault (andrea.a.perreault@vanderbilt.edu).

#### Materials Availability

This study did not generate new unique reagents.

#### Data and Code Availability

Unpublished custom code is available upon request from the Lead Contact. Summary of sequencing statistics can be found in Tables S1, S2, and S4. The accession number for the data reported in this paper is GEO SuperSeries GEO: GSE142006. Individual datasets can be found at GEO: GSE142003 (ChIP), GEO: GSE142004 (HiChIP), and GEO: GSE142005 (RNA-seq).

## Methods

All methods can be found in the accompanying Transparent Methods supplemental file.

## Supplemental Information

Document S1. Transparent Methods, Figures S1–S4 and Tables S1–S6
