# Supplementary material for: Erythropoietin Regulates Transcription and YY1 Dynamics in a Pre-established Chromatin Architecture
Source: iScience. 2020 Sep 20;23(10):101583. doi: 10.1016/j.isci.2020.101583 (PMC7559257; doi:10.1016/j.isci.2020.101583)
Supplement: Document S1. Transparent Methods, Figures S1–S4 and Tables S1–S6 [file mmc1.pdf]

iScience, Volume *23*

Supplemental Information

Erythropoietin Regulates Transcription and YY1 Dynamics in a Pre-established Chromatin Architecture

Andrea A. Perreault, Jonathan D. Brown, and Bryan J. Venters

Supplemental Information
Supplemental Figures and Legends

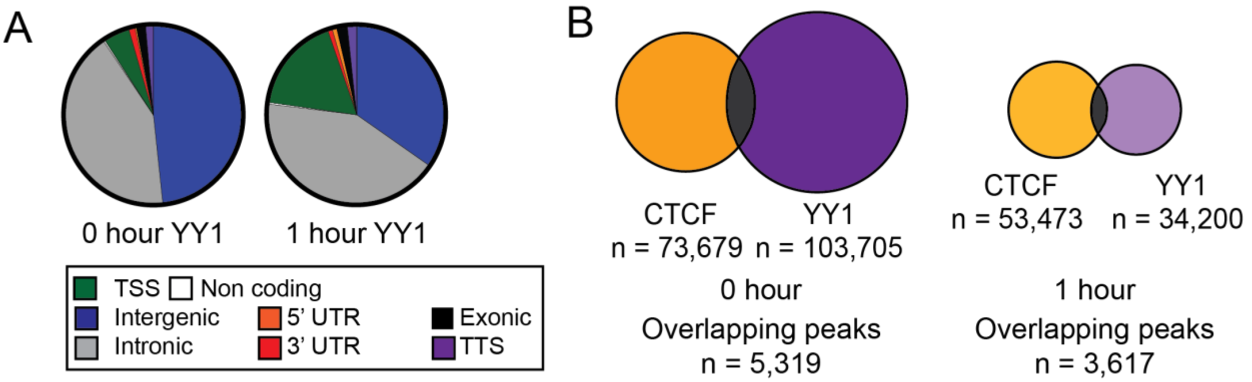

Figure S1. EPO dynamically regulates YY1 occupancy genome-wide, Related to Figure 3.
(A) YY1 binding locations in the genome.
(B) Comparison of CTCF and YY1 peak overlap before and after 1 hour EPO stimulation.

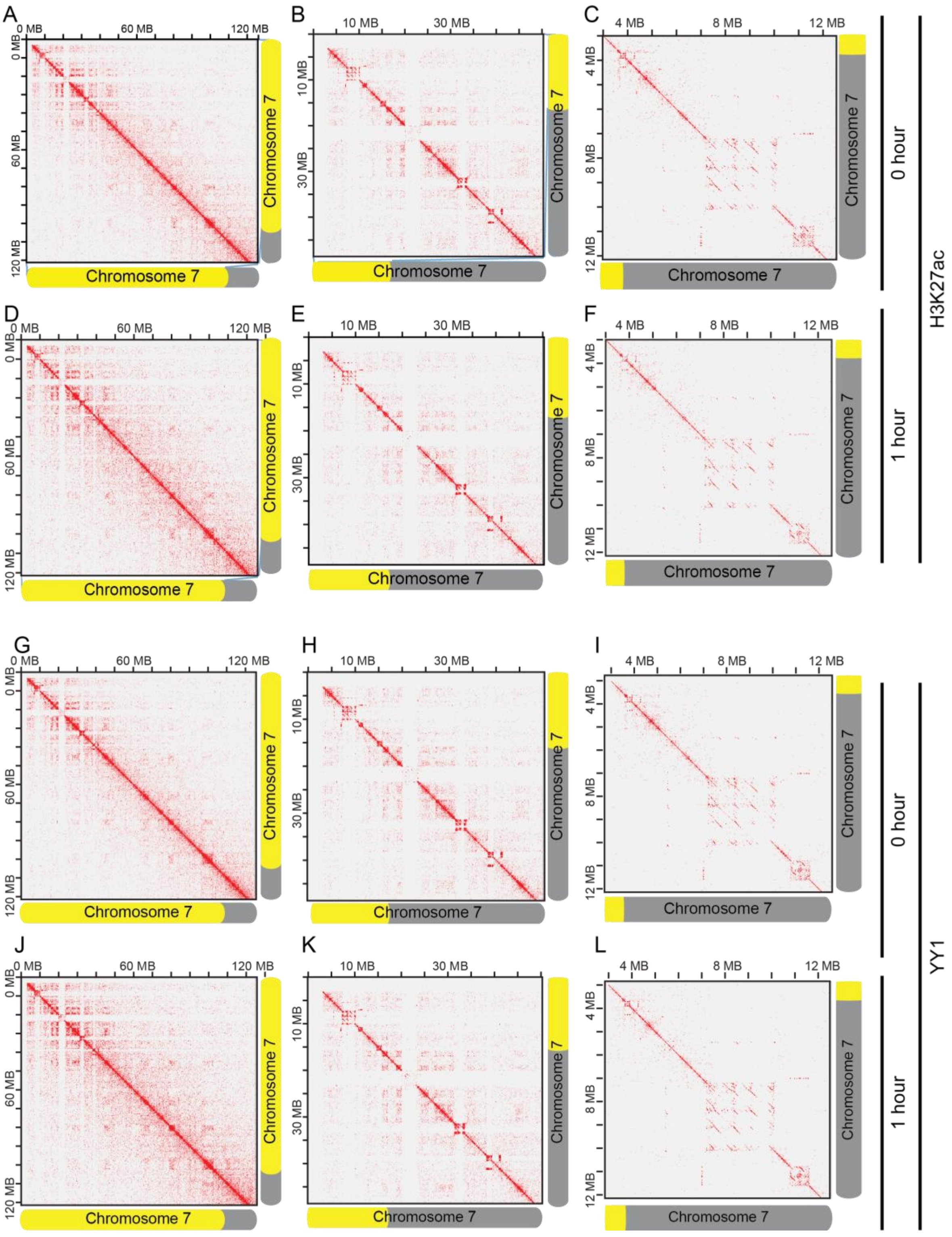

Figure S2. Representative chromatin contact maps for H3K27ac and YY1 HiChIP, Related to Figure 4.

Chromatin contacts mediated by H3K27ac at 0 hour EPO at (A) 250kb, (B) 100kb, and (C) 25kb resolutions.

Chromatin contacts mediated by H3K27ac at 1 hour EPO at (D) 250kb, (E) 100kb, and (F) 25kb resolutions.
Chromatin contacts mediated by YY1 at 0 hour EPO at (G) 250kb, (G) 100kb, and (I) 25kb resolutions.
Chromatin contacts mediated by YY1 at 1 hour EPO at (J) 250kb, (K) 100kb, and (L) 25kb resolutions.

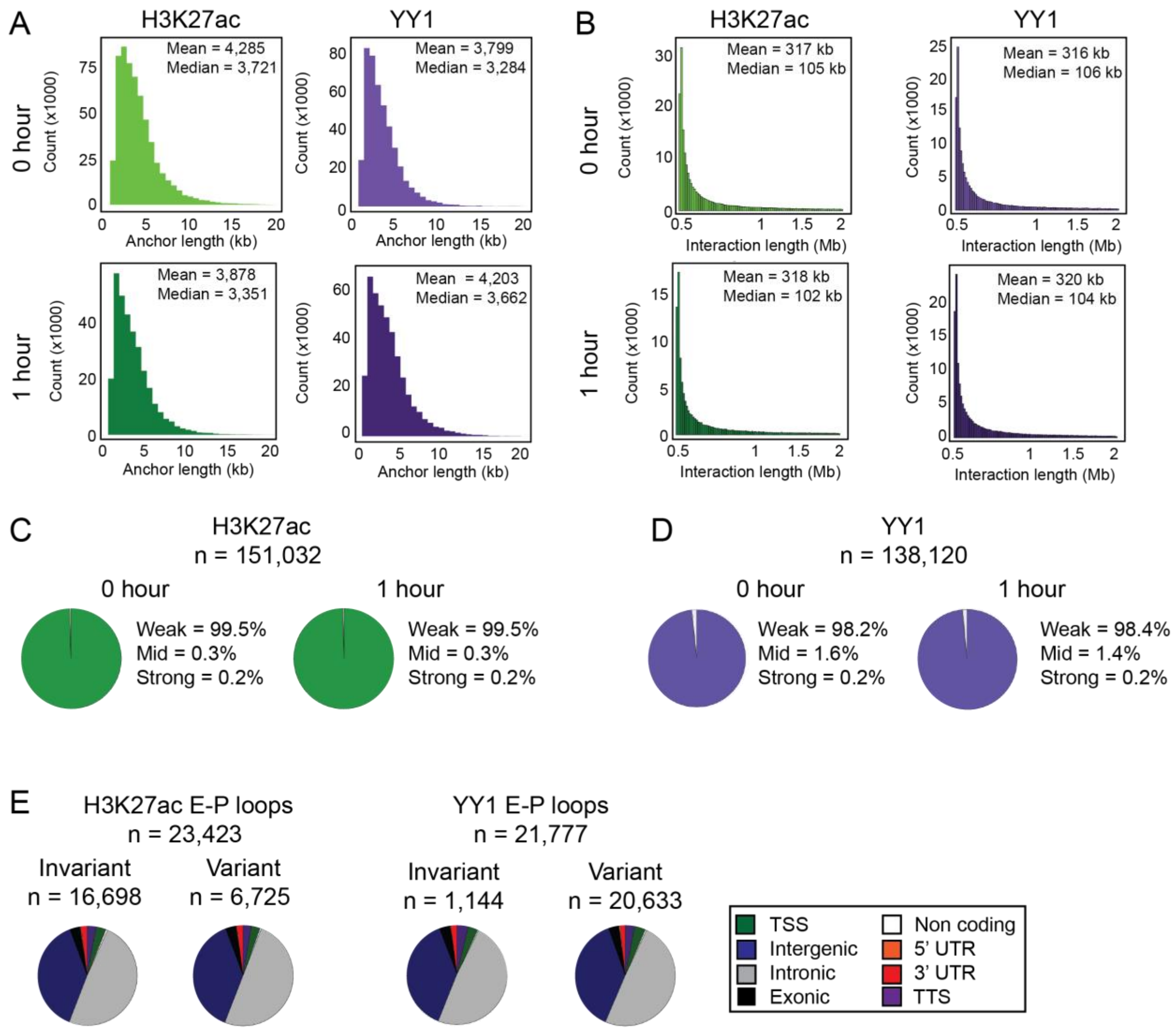

Figure S3. Characterization of chromatin loops mediated by H3K27ac and YY1, Related to Figure 4.

(A) Histogram showing the size distribution of anchors for HiChIP interactions in H3K27ac and YY1 libraries pre and post EPO stimulation.

(B) Histogram showing the size distribution of HiChIP interactions in H3K27ac and YY1 libraries pre and post EPO stimulation with log transformed x-axis.

(C) Fraction of weak (score < 5), moderate (score between 5 and 10), and strong (score > 10) H3K27ac chromatin interactions.

(D) Fraction of weak (score < 5), moderate (score between 5 and 10), and strong (score > 10) YY1 chromatin interactions.

(E) H3K27ac and YY1 enhancer-promoter (E-P) loops location in the genome.

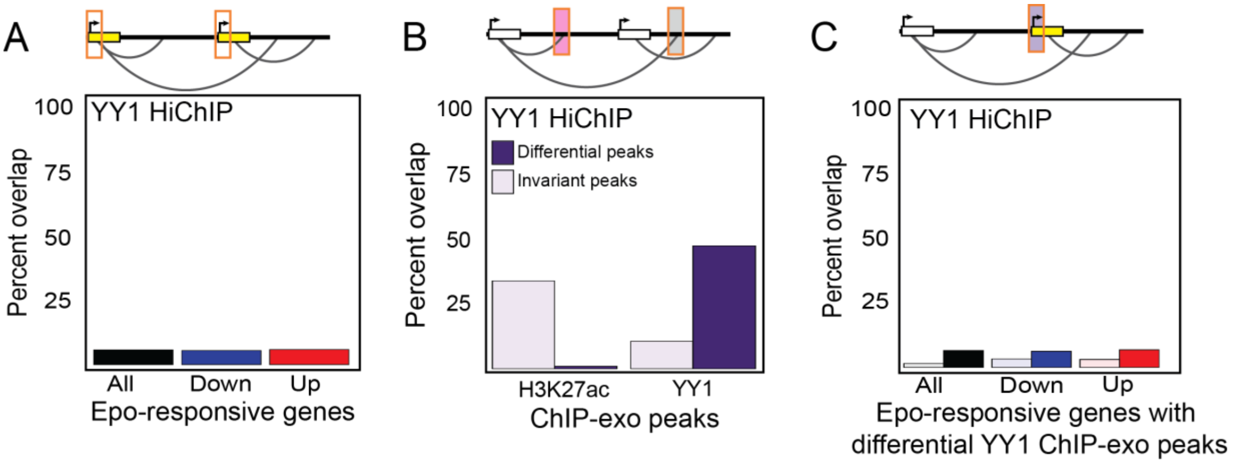

Figure S4. EPO regulates transcription in a pre-established chromatin conformation, Related to Figure 4.

(A) Proportion of interactions with promoters of EPO-responsive genes within YY1 HiChIP anchor regions.

(B) Proportion of interactions with differential H3K27ac or YY1 ChIP-exo peaks within anchor regions of YY1 HiChIP. Dark purple bars represent differential peaks TSS and light purple bars represent invariant peaks.

(C) Proportion of interactions with differential YY1 ChIP-exo peaks at promoters of EPO-responsive genes within YY1 HiChIP anchor regions. Dark bars represent EPO-responsive genes and light bars represent non-responsive genes.

Supplemental Tables

EPO (hr)	Total Reads	Reads Mapped	Mapping Rate	Unique Alignments	Unique Alignment Rate
0	51,086,988	46,916,946	92%	41,863,305	89%
	45,921,261	41,377,364	90%	37,200,184	90%
**TOTAL**	**97,008,249**	**88,294,310**		**79,063,489**	
1	43,417,043	39,605,767	91%	35,426,491	89%
	41,859,970	38,359,940	92%	34,287,633	89%
**TOTAL**	**85,277,013**	**77,965,707**		**69,714,124**	
4	45,650,667	42,564,629	93%	38,558,884	91%
	45,449,989	42,713,118	94%	38,620,867	90%
**TOTAL**	**91,100,656**	**85,277,747**		**77,179,751**	
12	48,350,536	44,651,856	92%	39,951,845	89%
	36,427,604	33,320,942	91%	29,800,144	89%
**TOTAL**	**84,778,140**	**77,972,798**		**69,751,989**	
24	49,732,518	43,101,597	87%	37,125,032	86%
	52,028,791	44,389,763	85%	38,266,645	86%
**TOTAL**	**101,761,309**	**87,491,360**		**75,391,677**	
36	40,613,328	33,511,866	83%	27,638,763	82%
	43,849,891	35,066,453	80%	28,300,690	81%
**TOTAL**	**84,463,219**	**68,578,319**		**55,939,453**	
Average Total Reads	**45,365,716**				
Average Mapped Reads		**40,465,020**			
Average Mapping Rate			**89%**		
Average Unique Alignments				**35,586,707**	
Average Unique Alignment Rate					**88%**

Table S1. RNA-seq sequencing statistics, Related to Figure 1.

Factor	Antibody	EPO (hr)	Total Reads	Uniquely Mapped	Unique Mapping Rate	Reads in Peaks	FRiP score
H3K27ac	ab4729	Previously published in Perreault, AA et al. (2017). Epo reprograms the epigenome of erythroid cells. *Experimental Hematology*. 51:47-62. Accession SRR4033061.					
RNA Pol II	sc-17798	0	23,599,539	18,845,128	80%		
			28,190,841	21,919,833	78%		
		**TOTAL**	**51,790,380**	**40,764,961**			
		1	25,380,733	19,181,745	76%		
			37,920,484	27,071,841	71%		
		**TOTAL**	**63,301,217**	**46,253,586**			
YY1	ab109237	0	49,303,713	32,145,330	65%		
			40,696,838	30,594,858	75%		
		**TOTAL**	**90,000,551**	**62,740,188**		8,881,135	0.141
		1	35,858,543	27,770,365	77%		
			43,771,372	32,777,027	75%		
		**TOTAL**	**79,629,915**	**60,547,392**		7,641,246	0.126
CTCF	07-729	0	53,987,299	45,182,628	84%		
			43,271,833	36,981,391	85%		
		**TOTAL**	**97,259,132**	**82,164,019**		40,119,395	0.488
		1	79,931,427	69,917,844	87%		
			37,329,553	30,956,490	83%		
		**TOTAL**	**117,260,980**	**100,874,334**		45,217,007	0.448
Average Total Reads			**41,603,515**				
Average Uniquely Mapped Reads				**32,778,707**			
Average Unique Mapping Rate					**77%**		

Table S2. ChIP-exo sequencing statistics, Related to Figure 2 and Figure 3.

Factor	Antibody	EPO (hr)	Pearson R Correlation
H3K27ac	ab4729	Previously published in Perreault, AA et al. (2017). Epo reprograms the epigenome of erythroid cells. *Experimental Hematology*. 51:47-62. Accession SRR4033061.	
RNA Pol II	sc-17798	0	0.80
		1	0.93
YY1	ab109237	0	0.97
		1	0.98
CTCF	07-729	0	0.97
		1	0.90

Table S3. ChIP-exo replicate correlation, Related to Figure 2 and Figure 3.

Factor	Antibody	EPO (hr)	Total Reads	Uniquely Mapped	Unique Mapping Rate
H3K27ac	ab4729	0	142,631,900	111,707,585	78%
			138,004,467	106,827,868	77%
		**TOTAL**	**280,636,367**	**218,535,453**	
		1	98,098,074	77,046,401	79%
			112,702,483	89,295,539	79%
		**TOTAL**	**210,800,557**	**166,341,940**	
YY1	ab109237	0	87,091,506	64,912,022	75%
			177,427,621	134,801,412	76%
		**TOTAL**	**264,519,127**	**199,713,434**	
		1	132,325,240	104,733,597	79%
			119,806,706	92,459,382	77%
		**TOTAL**	**252,131,946**	**197,192,979**	
Average Total Reads			**126,011,000**		
Average Uniquely Mapped Reads				**97,722,976**	
Average Unique Mapping Rate					**78%**

Table S4. HiChIP sequencing statistics, Related to Figure 4.

Factor	Antibody	EPO (hr)	Intra-chromosomal PETs	Long range interactions (5kb - 2Mb)	Percent intra-chromosomal PETs that are long range interactions	Chromatin loops (long range interactions between two ChIP anchors)	Percent of long range interactions that are chromatin loops
H3K27ac	ab4729	0	4,047,067	539,269	13%	110,994	21%
			3,007,741	236,596	8%	41,777	18%
		**TOTAL**	**7,054,808**	**775,865**		**152,771**	
		1	2,347,022	345,799	15%	33,399	10%
			2,684,603	222,089	8%	30,563	14%
		**TOTAL**	**5,031,625**	**567,888**		**63,962**	
YY1	ab109237	0	2,352,166	360,662	15%	34,018	9%
			4,594,705	389,653	8%	76,845	20%
		**TOTAL**	**6,946,871**	**750,315**		**110,863**	
		1	3,406,813	434,841	13%	72,080	17%
			3,096,245	243,649	8%	29,769	12%
		**TOTAL**	**6,503,058**	**678,490**		**101,849**	
Average intrachromosomal PETS			**3,192,045**				
Average long range interactions				**346,570**			
Average percent of long range interactions					**11%**		
Average chromatin loops						**53,681**	
Average percent of chromatin loops							**15%**

Table S5. HiChIP chromatin interaction statistics, Related to Figure 4.

Factor	Antibody	EPO (hr)	Pearson R Correlation
H3K27ac	ab4729	0	0.92
		1	0.98
YY1	ab109237	0	0.96
		1	0.98

Table S6. HiChIP chromatin interaction replicate correlation, Related to Figure 4.

Transparent Methods

REAGENT or RESOURCE	SOURCE	IDENTIFIER
**Antibodies**		
RNA Pol II	Santa Cruz	Cat# sc-17798
H3K27ac	Abcam	Cat# ab4729
YY1	Abcam	Cat# ab109237
CTCF	Millipore	Cat# 07-729
**Bacterial and Virus Strains**		
Friend Virus	Mark Koury lab	N/A
**Critical Commercial Assays**		
Qubit dsDNA High Sensitivity kit	Invitrogen	Cat# Q32854
Zymo DNA Clean and Concentrator kit	Zymo	Cat# D4003
Qiagen RNAeasy kit	Qiagen	Cat# 74104
NEBNext Ultra II Directional DNA library preparation kit	Illumina	Cat# E75530S
**Deposited Data**		
Raw and analyzed data	This study	GSE142006
Enhancer annotation	(Perreault et al., 2017)	SRP082181
Mouse reference genome, NCBI build GRCm38/mm10	Genome Reference Consortium	https://www.ncbi.nlm.nih.gov/grc/mouse
**Experimental Models: Organisms/Strains**		
Mouse: Female BALB/cJ, 12 weeks	The Jackson Laboratory	
**Software and Algorithms**		
BWA-MEM	(Li and Durbin, 2010)	http://bio-bwa.sourceforge.net
Samtools	(Li et al., 2009)	http://samtools.sourceforge.net
HOMER	(Heinz et al., 2010)	http://homer.ucsd.edu/homer/
deepTOOLS	(Ramirez et al., 2014)	https://deeptools.readthedocs.io/en/develop/
BEDTools	(Quinlan, 2014)	https://bedtools.readthedocs.io/en/latest/
IGV	(Robinson et al., 2011)	https://software.broadinstitute.org/software/igv/
WashU Epigenome Browser	(Zhou et al., 2011)	http://epigenomegateway.wustl.edu
HiC-Pro	(Servant et al., 2015)	https://github.com/nservant/HiC-Pro

hichipper	(Lareau and Aryee, 2018b)	https://github.com/aryeelab/hichipper
Juicer	(Durand et al., 2016)	https://github.com/aidenlab/juicer/wiki/Juicer-Tools-Quick-Start
diffloop	(Lareau and Aryee, 2018a)	https://bioconductor.org/packages/release/bioc/html/diffloop.html
TopHat	(Trapnell et al., 2009)	https://ccb.jhu.edu/software/tophat/index.shtml
Cufflinks	(Trapnell et al., 2012)	http://cole-trapnell-lab.github.io/cufflinks/
cummeRbund		http://compbio.mit.edu/cummeRbund/ R package version 2.26.0
R		https://www.r-project.org
edgeR	(Robinson et al., 2010)	https://bioconductor.org/packages/release/bioc/html/edgeR.html
MACS	(Zhang et al., 2008)	https://github.com/taoliu/MACS

EXPERIMENTAL MODEL AND SUBJECT DETAILS

Isolation of Proerythroblasts from FVA infected mice

Highly purified proerythroblasts were obtained from spleens of mice infected with the Friend virus as previously described(Sawyer et al., 1987, Koury et al., 1984), with the following modifications. All animal procedures were performed in compliance with and approval from the Vanderbilt Division of Animal Care (DAC) and Institutional Animal Care and Use Committee (IACUC). Female BALB/cJ mice (12 weeks old, Jackson Laboratories) were infected via intraperitoneal injection of ~10_4 spleen focus-forming units of Anemia-inducing strain of the Friend virus (FVA). At 13 to 15 days post-infection, the mice were sacrificed and spleens removed. The spleens were homogenized to a single cell suspension by passing the minced spleens through a sterile 100 micron nylon mesh filter into sterile solution of 0.2% bovine serum albumin (BSA) in 1x PBS. The filtrate was then repeatedly pipetted to ensure a single cell suspension. The homogenized spleen cells were size-separated by gravity sedimentation for 4 hours at 4°C in a continuous gradient of 1% to 2% deionized BSA.

The sedimentation apparatus consisted of a 25cm diameter sedimentation chamber containing a 2.4L BSA gradient, two BSA gradient chambers containing 1.2L 1% and 2% deionized BSA in 1x PBS, and a cell loading chamber (ProScience Inc.) containing the 50ml cell suspension. After 4 hour sedimentation, cells were collected in 50ml fractions, with proerythroblasts typically enriched in fractions 5-20 of 24 total fractions. Typically about 10^9 proerythroblasts were obtained from the separation of 10^{10} nucleated spleen cells (6-7g spleen weight) across three 25cm sedimentation chambers.

Cell Culture Conditions
To study the effects of erythropoietin (EPO) on terminal erythroid differentiation, FVA-derived proerythroblasts were cultured at 10^6 cells/ml in Iscove-modified Dulbecco medium (IMDM, Life Technologies #12440043), 30% heat-inactivated fetal bovine serum (Gibco, 26140-079), 1% Penicillin-Streptomycin (Gibco #15140-122), 10% deionized BSA, and 100uM alpha-thioglycerol (MP Biomedicals #155723). Terminal erythroid differentiation of purified proerythroblasts was induced by the addition of 0.4 U/ml human recombinant EPO (10kU/ml Epogen by Amgen, NDC 55513-144-10) to media. At the desired times after the addition of EPO, cells were crosslinked by the addition of 1% formaldehyde for 10 minutes for ChIP analysis and 2% formaldehyde for 20 minutes for HiChIP analysis. Crosslinking was then quenched by the addition of 125mM glycine. Crosslinked cells were collected by centrifugation for 5 minutes at 1,000g at 4°C, washed once with 1x PBS, flash frozen in liquid nitrogen, and stored at -80°C until used. For RNA-seq, cells were removed from culture before crosslinking. Samples were spun for 5 minutes at 1,000g at 4°C and the supernatant was aspirated. Pellets were flash frozen in liquid nitrogen and stored at -80°C until used.

EXPERIMENTAL DESIGN

All experiments were replicated. No aspect of the study was done blinded. Sample size was not predetermined.

HiChIP
HiChIP was performed as described(Mumbach et al., 2016) with a few modifications.
In Situ Contact Generation
50 million cell pellets were resuspended in 2.5ml ice cold Hi-C Lysis Buffer (10mM Tris HCl, 10mM NaCl, 0.2% NP-40, 1X protease inhibitors (Roche, 04693124001)) and split into 10 million cell amounts. Samples were incubated at 4°C for 30 minutes with rotation. Nuclei were pelleted by centrifugation at 2,500g for 5 minutes at 4°C and washed once with 500ul of ice cold Hi-C Lysis Buffer. After removing supernatant, nuclei were resuspended in 100ul of 0.5% SDS and incubated at 62°C for 10 minutes. SDS was quenched by adding 285ul water and 50ul 10% Triton X-100. Samples were vortexed and incubated for 15 minutes at 37°C. After the addition of 50ul of 10X NEBBuffer 2 (NEB, B7002) and 1ul of MboI restriction enzyme (NEB, R0147), chromatin was digested at 37°C for 1 hour at 700rpm on Thermomixer. Following digestion, MboI enzyme was heat inactivated by incubating the nuclei at 62°C for 20

minutes. To fill in the restriction fragment overhangs and mark the DNA ends with biotin, 52ul of fill-in master mix, containing 15ul of 1mM biotin-dATP (Jena BioScience, NU-835-BIO14-L), 1.5ul of 10mM dCTP (NEB, N044_S), 1.5ul of 10mM dGTP (NEB, N044_S), 1.5ul of 10mM dTTP (NEB, N044_S), and 10ul of 5 U/ul DNA Polymerase I, Large (Klenow) Fragment (NEB, M0210), was added and the tubes were incubated at 37°C for 1 hour at 700rpm on Thermomixer. Proximity ligation was performed by addition of 948ul of ligation master mix, containing 150ul of 10X NEB T4 DNA ligase buffer (NEB, B0202), 125ul of 10% Triton X-100, 15ul of 10 mg/mL BSA (NEB, B9000), 10ul of 400 U/mL T4 DNA ligase (NEB, M0202), and 648ul of water, and incubation at room temperature for 4 hours with rotation.

Sonication and Chromatin Immunoprecipitation

After proximity ligation, nuclei were pelleted by centrifugation at 2500g for 5 minutes and resuspended in 880ul Nuclear Lysis Buffer (50mM Tris HCl, 10mM EDTA, 1% SDS, 1X protease inhibitors (Roche, 04693124001)). Samples were vortexed and nuclei were sonicated with a Bioruptor (Diagenode) for 10 minutes on the low setting to solubilize chromatin. Sonicated chromatin was clarified by centrifugation at 16,100g for 15 min at 4°C and supernatant from 10 million cell samples are pooled to a total of 50 million cells. Sample was diluted with 2X ChIP Dilution Buffer (0.01% SDS, 1.1% Triton X-100, 1.2mM EDTA, 16.7mM Tris HCl, 167mM NaCl). 300ul Protein A beads (Thermo, 21348) were washed in 2ml ChIP Dilution Buffer and resuspended in 250ul ChIP Dilution Buffer. Beads were added to 50 million cell sample and incubated at 4°C for 1 hour with rotation. Beads were then separated on a magnetic rack and supernatant was transferred to a new tube. 10ug of antibody for Pol II (Santa Cruz, sc-17798), H3K27ac (Abcam, ab4729), or YY1 (Abcam, ab109237) were added to the tube. Samples were incubated overnight at 4°C with rotation. The next day, 300ul Protein A beads were washed in 2ml ChIP Dilution Buffer and resuspended in 500ul ChIP Dilution Buffer. Beads were added to 50 million cell sample with antibody and incubated at 4°C for 2 hours with rotation. Beads were then separated on a magnetic rack and washed three times with 750ul Low Salt Wash Buffer (0.1% SDS, 1% Triton X-100, 2mM EDTA, 20mM Tris HCl, 150mM NaCl), three times with 750ul High Salt Wash Buffer (0.1% SDS, 1% Triton X-100, 2mM EDTA, 20mM Tris HCl, 500mM NaCl), and three times with 750ul LiCl Wash Buffer (10mM Tris HCl, 250mM LiCl, 1% NP-40, 1% Na-Doc, 1mM EDTA).

DNA Elution and Reverse Crosslinking

Beads were then resuspended in 200ul of DNA Elution Buffer (50mM $NaHCO_3$, 1% SDS), which is made fresh, and incubated at room temperature for 10 minutes with rotation, followed by 37°C for 3 minutes at 700rpm. Samples were placed on a magnetic rack and supernatant transferred to a new tube. This was repeated once more. 10ul of Proteinase K (Roche, 03115828001) was added to each tube and samples were incubated at 55°C for 45 minutes at 700rpm, followed by 67°C for 1.5 hours at 700rpm. DNA was then purified using Zymo DNA Clean and Concentrator (Zymo, D4003) according to manufacturer's protocol and eluted in 10ul water. The amount of eluted DNA was quantified by Qubit dsDNA HS kit (Invitrogen, Q32854).

Biotin Capture and Sequencing Preparation

25ul of Streptavidin C-1 beads (Invitrogen, 65001) were washed with 1ml Tween Wash Buffer (5MM Tris HCl, 0.5mM EDTA, 1M NaCl, 0.05% Tween-20) and resuspended in

10ul of 2X Biotin Binding Buffer (10mM Tris HCl, 1mM EDTA, 2M NaCl). 10ul of bead mixture was added to 50ng of purified DNA for each sample, incubating at room temperature for 15 minutes, agitating every 5 minutes. After capture, beads were separated with a magnet and the supernatant was discarded. Beads were then washed twice with 500ul of Tween Wash Buffer, incubating at 55°C for 2 minutes at 700rpm. Beads were washed with 100ul 1X TD Buffer (diluted from 2X TD Buffer (20mM Tris HCl, 10mM $MgCl_2$, 20% Dimethylformamide)). Beads were resuspended in 50ul of master mix, containing 25ul 2X TD Buffer, 2.5ul Tn5 Tagment DNA enzyme (Illumina, 15027865), and 22.5ul water. Samples were incubated at 55°C for 10 minutes at 700rpm. Beads were separated on a magnet and supernatant was discarded. Beads were washed with 750ul of 50mMEDTA at 50°C for 30 minutes, washed twice with 750ul of 50mMEDTA at 50°C for 3 minutes each, then washed twice with 750ul of Tween Wash Buffer at 55°C for 2 minutes each, and finally washed once with 750uL of 10mM Tris HCl pH 7.5. Beads were separated on a magnet and supernatant was discarded.

PCR and Size Selection

To generate the sequencing library, PCR amplification of the tagmented DNA was performed while the DNA is still bound to the beads. Beads were resuspended in a PCR master mix, consisting of 36ul water, 1.25 unique Nextera Ad2.X primer, 10ul Phusion HF 5X buffer (NEB, E0553), 1ul 10mM dNTPs, 1.25ul universal Nextera Ad1 primer, and 0.5ul Phusion DNA Polymerase (NEB, E0553). DNA was amplified with 8 cycles of PCR. After PCR, beads were separated on a magnet and the supernatant containing the PCR amplified library was transferred to a new tube, purified using the Zymo DNA Clean and Concentrator (Zymo D4003) kit according to manufacturer's protocol and eluted in 52ul water. Purified HiChIP libraries were size selected to 300-700 basepairs using a double size selection with AMPure XP beads (Beckman Coulter, A68831). HiChIP libraries were paired-end sequenced on an Illumina NextSeq500 with reads 75 nucleotides in length.

Chromatin Immunoprecipitation with Lambda Exonuclease Digestion (ChIP-exo)

With the following modifications, ChIP-exo was performed as previously described(Perreault and Venters, 2016, Rhee and Pugh, 2011) with chromatin extracted from 50 million cells, ProteinG MagSepharose resin (GE Healthcare), and 10ug of antibody directed against Pol II (Santa Cruz, sc-17798), YY1 (Abcam, ab109237), or CTCF (Millipore, 07-729). First, formaldehyde crosslinked cells were lysed with buffer 1 (50mM HEPES–KOH pH 7.5, 140mM NaCl, 1 mM EDTA, 10% Glycerol, 0.5% NP-40, 0.25% Triton X-100), washed once with buffer 2 (10mM Tris HCL pH 8, 200mM NaCl, 1mM EDTA, 0.5mM EGTA), and the nuclei lysed with buffer 3 (10mM Tris HCl pH 8, 100mM NaCl, 1mM EDTA, 0.5mM EGTA, 0.1% Na–Deoxycholate, 0.5% *N*-lauroylsarcosine). All cell lysis buffers were supplemented with fresh EDTA-free complete protease inhibitor cocktail (CPI, Roche #11836153001). Purified chromatin was sonicated with a Bioruptor (Diagenode) to obtain fragments with a size range between 100 and 500 base pairs. Triton X-100 was added to extract at 1% to neutralize sarcosine. Insoluble chromatin debris was removed by centrifugation, and sonication extracts stored at -80°C until used for ChIP analysis. Libraries were sequenced using an

Illumina NextSeq500 sequencer as single-end reads 75 nucleotides in length on high output mode.

RNA-seq
RNA was isolated using the Qiagen RNAeasy kit (Qiagen, 74104) per manufacturer's instructions. Stranded polyA selected libraries were prepared using NEBNext PolyA mRNA isolation standard protocol, NEBNext rRNA Depletion standard protocol, and finally NEBNext Ultra II Directional DNA library preparation kit (Illumina, E75530S) per manufacturer's protocol. PCR amplified RNA-seq libraries were size selected using AMPure XP beads (Beckman Coulter, A68831). RNA-seq libraries were subjected to 75 basepair single end sequencing on Illumina NextSeq500 sequencer.

QUANTIFICATION AND STATISTICAL ANALYSIS

HiChiP data analysis

Alignment
HiChIP library sequence reads were aligned to the mouse mm10 reference genome using HiC-Pro(Servant et al., 2015) with the following options in the configuration file:

BOWTIE2_OPTIONS = --very-sensitive --end-to-end –reorder
LIGATION_SITE = GATCGATC
GET_ALL_INTERACTION_CLASSES = 1
GET_PROCESS_SAM = 1
RM_SINGLETON = 1
RM_MULTI = 1
RM_DUP = 0

Use of replicates
Biological replicates were run through HiC-Pro in parallel. Replicate correlation was assessed after HiC-Pro processing. Specifically, .allValidPairs files were compared using Pearson's correlation test. R values can be found in Supplemental Table 6.

Chromatin interaction identification
Hichipper(Lareau and Aryee, 2018b) was applied to HiC-Pro output files to identify high confidence chromatin contacts using EACH, ALL peak finding settings. Interaction calls for each replicate are considered individually for loop analysis and annotation in hichipper. The quickAssoc and annotateLoops functions in the diffloop R package(Lareau and Aryee, 2018a) were used to find differential loops and annotate epigenetic features, respectively. Enhancers were denoted as the intersection of H3K4me1 and H3K27ac peaks (previously published data) and promoters were identified using the getMouseTSS function.

HiChIP display
To visualize chromatin interactions identified using HiChIP, the –make-ucsc option was added when analyzing the data using hichipper(Lareau and Aryee, 2018b).

ChIP-exo data analysis

Alignment

ChIP-exo library sequence reads were aligned to the mouse mm10 reference genome using BWA-MEM algorithm(Li and Durbin, 2010) using default parameters. The resulting bam files were first sorted using the Samtools Sort function(Li et al., 2009), and then bam index files were generated using the Samtools Index function(Li et al., 2009).

Peak calling

ChIP-exo peaks were annotated and quantified using the Hypergeometric Optimization of Motif EnRichment (HOMER) suite(Heinz et al., 2010). Briefly, bam files were converted to tag directories using the makeTagDirectory function with the –genome, –checkGC, and –format options. The findPeaks function was used to identify ChIP peaks using –o auto and –style gro-seq or factor for Pol II or CTCF/YY1 libraries, respectively. To quantify and normalize tags to RPKM, the analyzeRepeats function was used with the –rpkm, –count genes, –strand both, –condenseGenes, and –d options.

Use of replicates

Replicate correlation was assessed after peak calling. Specifically, RPKM calculated from analyzeRepeats function were plotted using scatterplot compared using Pearson's correlation test. R values can be found in Supplementary Table 3. Replicates were merged for final analyses presented in the manuscript.

Heatmaps

bigWig files for CTCF and YY1 libraries were generated using the deepTools bamCoverage function(Ramirez et al., 2014). To create aligned heatmaps, first a matrix was generated using the computeMatrix function with the following options: reference-point –S, –a 2000, –b 2000, -–referencePoint center, –verbose, –missingDataAsZero, and –p max/2. Then, the heatmap was created using the plotHeatmap function with the following options: –verbose and –sortRegions descend.

ChIP-exo display

Raw sequencing tags were smoothed (20 basepair bin, 100 basepair sliding window) and normalized to reads per kilobase per million (RPKM) using deepTOOLS(Ramirez et al., 2014) and visualized with Integrative Genomics Viewer (IGV)(Robinson et al., 2011).

RNA-seq data analysis

RNA-seq alignment, transcript assembly, and differential expression

RNA-seq library sequence reads were aligned to the mouse mm10 reference genome using TopHat(Trapnell et al., 2009) using default parameters. Cufflinks(Trapnell et al., 2012) was used to assemble transcripts and quantify expression of transcripts. Cuffmerge(Trapnell et al., 2012) merges all transcript assemblies to create a single merged transcriptome annotation for final analyses. The program conducts multiple hypothesis correction and calculates an adjusted FDR q-value.

Use of replicates

Replicates are both used as input for the cufflinks and cuffmerge programs described above.

RNA-seq display

CummeRbund visualizes RNA-seq data analyzed using cufflinks.

Definition of regulatory regions

Throughout the manuscript multiple analyses rely on overlaps with different regulatory regions, namely enhancers and promoters. Here we explain how these regulatory regions were defined.

Promoters are defined here as the comprehensive list of annotated transcription start sites (TSS) in the mm10 mouse genome from UCSC.

Enhancers are defined here as regions of the genome marked by H3K4me1 and H3K27ac. This group is further supported by enhancer identified using ChromHMM in (Perreault et al., 2017).

Definition of chromatin features

Throughout the manuscript multiple analyses rely on overlaps with different chromatin features. Here we explain how these features were defined.

HiChIP anchors, as identified by the hichipper analysis pipeline, are the regions between restriction enzyme motifs that contain a ChIP peak for the factor of interest by extending ChIP peaks to the edges of the restriction fragment. As a consequence of this computational definition, HiChIP anchors typically span a wide range of lengths. In the present study, we use anchor as a broad term to define the endpoints of a HiChIP loop.

HiChIP loops are defined as the distance between two ends of a chromatin interaction called anchors, which are identified in hichipper. These loops have a specific score, which is the number of paired-end tags (PETs) that support the interaction. In this study, we separate loops into 3 categories (weak, moderate, and strong) based on the interquartile range of the loop scores determined by diffloop.

Invariant loops are chromatin interactions that satisfy two criteria. First, these loops have a fold change in score (as calculated by diffloop) to be between -2 and 2. A fold change of +/-2 is commonly used in the literature to separate variant and invariant features and was used here as a continuation of our previously published work. Second, FDR > 0.1 (as calculated by diffloop). Usually FDR < 0.1 would subset the group of chromatin loops that are significantly different between two conditions. Therefore, the complement of this group is the subset of chromatin loops that are not significantly different.

Supplemental References

DURAND, N. C., SHAMIM, M. S., MACHOL, I., RAO, S. S., HUNTLEY, M. H., LANDER, E. S. & AIDEN, E. L. 2016. Juicer Provides a One-Click System for Analyzing Loop-Resolution Hi-C Experiments. *Cell Syst,* 3**,** 95-8.

HEINZ, S., BENNER, C., SPANN, N., BERTOLINO, E., LIN, Y. C., LASLO, P., CHENG, J. X., MURRE, C., SINGH, H. & GLASS, C. K. 2010. Simple combinations of lineage-determining transcription factors prime cis-regulatory elements required for macrophage and B cell identities. *Mol Cell,* 38**,** 576-89.

KOURY, M. J., SAWYER, S. T. & BONDURANT, M. C. 1984. Splenic erythroblasts in anemia-inducing Friend disease: a source of cells for studies of erythropoietin-mediated differentiation. *Journal of cellular physiology,* 121**,** 526-32.

LAREAU, C. A. & ARYEE, M. J. 2018a. diffloop: a computational framework for identifying and analyzing differential DNA loops from sequencing data. *Bioinformatics,* 34**,** 672-674.

LAREAU, C. A. & ARYEE, M. J. 2018b. hichipper: a preprocessing pipeline for calling DNA loops from HiChIP data. *Nat Methods,* 15**,** 155-156.

LI, H. & DURBIN, R. 2010. Fast and accurate long-read alignment with Burrows-Wheeler transform. *Bioinformatics,* 26**,** 589-95.

LI, H., HANDSAKER, B., WYSOKER, A., FENNELL, T., RUAN, J., HOMER, N., MARTH, G., ABECASIS, G., DURBIN, R. & GENOME PROJECT DATA PROCESSING, S. 2009. The Sequence Alignment/Map format and SAMtools. *Bioinformatics,* 25**,** 2078-9.

MUMBACH, M. R., RUBIN, A. J., FLYNN, R. A., DAI, C., KHAVARI, P. A., GREENLEAF, W. J. & CHANG, H. Y. 2016. HiChIP: efficient and sensitive analysis of protein-directed genome architecture. *Nat Methods,* 13**,** 919-922.

PERREAULT, A. A., BENTON, M. L., KOURY, M. J., BRANDT, S. J. & VENTERS, B. J. 2017. Epo reprograms the epigenome of erythroid cells. *Exp Hematol*.

PERREAULT, A. A. & VENTERS, B. J. 2016. The ChIP-exo Method: Identifying Protein-DNA Interactions with Near Base Pair Precision. *J Vis Exp*.

QUINLAN, A. R. 2014. BEDTools: The Swiss-Army Tool for Genome Feature Analysis. *Curr Protoc Bioinformatics,* 47**,** 11 12 1-11 12 34.

RAMIREZ, F., DUNDAR, F., DIEHL, S., GRUNING, B. A. & MANKE, T. 2014. deepTools: a flexible platform for exploring deep-sequencing data. *Nucleic Acids Res,* 42**,** W187-91.

RHEE, H. S. & PUGH, B. F. 2011. Comprehensive genome-wide protein-DNA interactions detected at single-nucleotide resolution. *Cell,* 147**,** 1408-19.

ROBINSON, J. T., THORVALDSDOTTIR, H., WINCKLER, W., GUTTMAN, M., LANDER, E. S., GETZ, G. & MESIROV, J. P. 2011. Integrative genomics viewer. *Nat Biotechnol,* 29**,** 24-6.

ROBINSON, M. D., MCCARTHY, D. J. & SMYTH, G. K. 2010. edgeR: a Bioconductor package for differential expression analysis of digital gene expression data. *Bioinformatics,* 26**,** 139-40.

SAWYER, S. T., KOURY, M. J. & BONDURANT, M. C. 1987. Large-scale procurement of erythropoietin-responsive erythroid cells: assay for biological activity of erythropoietin. *Methods Enzymol,* 147**,** 340-52.

SERVANT, N., VAROQUAUX, N., LAJOIE, B. R., VIARA, E., CHEN, C. J., VERT, J. P., HEARD, E., DEKKER, J. & BARILLOT, E. 2015. HiC-Pro: an optimized and flexible pipeline for Hi-C data processing. *Genome Biol,* 16**,** 259.

TRAPNELL, C., PACHTER, L. & SALZBERG, S. L. 2009. TopHat: discovering splice junctions with RNA-Seq. *Bioinformatics,* 25**,** 1105-11.

TRAPNELL, C., ROBERTS, A., GOFF, L., PERTEA, G., KIM, D., KELLEY, D. R., PIMENTEL, H., SALZBERG, S. L., RINN, J. L. & PACHTER, L. 2012. Differential gene and transcript expression analysis of RNA-seq experiments with TopHat and Cufflinks. *Nat Protoc,* 7**,** 562-78.

ZHANG, Y., LIU, T., MEYER, C. A., EECKHOUTE, J., JOHNSON, D. S., BERNSTEIN, B. E., NUSBAUM, C., MYERS, R. M., BROWN, M., LI, W. & LIU, X. S. 2008. Model-based analysis of ChIP-Seq (MACS). *Genome Biol,* 9**,** R137.

ZHOU, X., MARICQUE, B., XIE, M., LI, D., SUNDARAM, V., MARTIN, E. A., KOEBBE, B. C., NIELSEN, C., HIRST, M., FARNHAM, P., KUHN, R. M., ZHU, J., SMIRNOV, I., KENT, W. J., HAUSSLER, D., MADDEN, P. A., COSTELLO, J. F. & WANG, T. 2011. The Human Epigenome Browser at Washington University. *Nat Methods,* 8**,** 989-90.
